# The Grandmother Effect: A Qualitative Study Examining How We Pass Down Values of Black Womanhood

**DOI:** 10.1093/geroni/igaf122.3311

**Published:** 2025-12-31

**Authors:** Tanya Burgess

**Affiliations:** Western Kentucky University, Bowling Green, Kentucky, United States

## Abstract

This qualitative study explores the lived experiences of Black women aged 55 and above residing in Maricopa County, Arizona, with a focus on their perspectives of care, social connections, and intergenerational caregiving norms and values. Through in-depth interviews lasting 60 to 90 minutes, participants shared their narratives on how social engagement and caregiving practices shape their sense of belonging and well-being. Findings revealed that all participants attributed their caregiving approach and social interactions to deeply held cultural values, emphasizing mentorship, service, and intergenerational guidance. These values were largely learned through strong relationships with older Black women, particularly grandmothers. The study highlights that Black women’s sense of community is reinforced not solely through mentorship but through structured intergenerational support systems that foster belonging and reduce the pressures associated with the “superwoman” schema. The implications of this study underscore the need for community interventions, services, and policies that move beyond traditional mentorship models. Instead, efforts should be directed toward creating spaces where Black women of all ages can engage in reciprocal care and knowledge-sharing without reinforcing harmful narratives of self-sacrifice. By acknowledging and strengthening intergenerational ties, communities can better support the caregiving trajectories of Black women, ensuring holistic and sustainable well-being across generations.

